# Assortative Mating Is a Natural Consequence of Heritable Variation in Preferences and Preferred Traits

**DOI:** 10.1177/09567976251365900

**Published:** 2025-09-04

**Authors:** Kaitlyn T. Harper, Brendan P. Zietsch

**Affiliations:** Centre for Psychology and Evolution, School of Psychology, University of Queensland

**Keywords:** assortative mating, mate choice, genetic correlations, agent-based modeling, evolutionary psychology

## Abstract

Assortative mating—the tendency to choose partners similar to oneself—is a ubiquitous phenomenon in mate choice. Despite numerous proposed explanations, a parsimonious mechanism has been overlooked: When individuals choose mates on the basis of heritable traits and preferences, offspring inherit a trait and the corresponding preference from each parent, creating genetic correlations that link having a trait to preferring that same trait. We evaluated this mechanism with an agent-based model simulating 100 generations in which agents, with traits and preferences each uniquely determined by 40 loci, chose reproductive partners based on preferences. Genetic correlations formed between preferences and preferred traits, as well as between partner traits (i.e., assortative mating), demonstrating that heritable variation in preferences and preferred traits is sufficient to drive assortative mating. We presented a toy model here, so we cannot speak to the robustness of such genetic correlations or to the relative explanatory power of this mechanism over others.

Choosing a romantic partner is one of the most pivotal decisions in a person’s life; it has lasting implications for mental and physical well-being (Robles et al., 2014) and a substantial economic impact. Given the profound impact on life satisfaction, it is no surprise that mate choice is a prominent aspect of human behavior and culture. A ubiquitous pattern in mate choice is *assortative mating*, where individuals tend to select partners similar to themselves. This effect is well-documented in humans (Horwitz et al., 2023; Luo, 2017) and other species (Jiang et al., 2013). In humans, assortative mating has been extensively observed in physical, personality, and demographic characteristics (see Horwitz et al., 2023, for a review).

The mechanisms driving assortative mating warrant close attention because of its substantial impact across multiple domains. Specifically, assortative mating on socioeconomic traits can intensify resource disparities (Harpending & Cochran, 2015), leading to a deeper divide between social classes (Blossfeld, 2009). Beyond socioeconomic implications, assortative mating also impacts the genomic structure of populations, fostering genetic clustering within subgroups and pushing populations toward genetic extremes (Abdellaoui et al., 2015). This process can concentrate genetic risk factors for health issues like psychiatric conditions and obesity, creating clusters of high-risk populations (Cabrera-Mendoza et al., 2024; Speakman et al., 2007).

Further, the effects of assortative mating introduce challenges to genetic studies themselves. By violating the assumption of random mating, assortative mating can deflate heritability estimates in classic twin designs, leading to misinterpretations of the genetic and developmental basis of traits (Willoughby et al., 2023). Even large-scale genome-wide association studies (GWAS) can be impacted by assortative mating on traits like education and ancestry. These patterns can distort measures of genetic relatedness, bias heritability estimates, and introduce unexpected genetic correlations, all of which complicate the interpretation of genetic influences on traits (Abdellaoui et al., 2022; Hugh-Jones et al., 2016; Sebro et al., 2017).

A variety of mechanisms have been proposed to explain assortative mating (see Versluys et al., 2021, and Luo, 2017, for reviews). Some mechanisms involve social dynamics such as population stratification and social homogamy (Abdellaoui et al., 2014), and mating markets (Kalick & Hamilton, 1986; Xie et al., 2015). Other mechanisms suggest that assortative mating provides evolutionary fitness benefits through enhanced compatibility, both socially (Ihle et al., 2015) and biologically (Tregenza & Wedell, 2000), through increased genetic relatedness to offspring (Rushton, 1989), and when traits at extreme values are advantageous (Jiang et al., 2013). Each of these processes may apply plausibly to some traits and not others. Here, we present an overlooked genetic mechanism that can explain the ubiquity of assortative mating: Assortative mating is a natural consequence of heritable variation in preferences and preferred traits.

When individuals choose partners on the basis of heritable preferences and preferred traits, genetic correlations form between individuals’ traits and corresponding preferences because offspring inherit both the trait from one parent and the corresponding preference from the other. These genetic correlations between preferences and preferred traits mean that individuals are likely to choose partners with similar traits to their own, because preferring a trait is associated with having that same trait. For example, if an individual has a preference for tallness, and consequently chooses to mate with a tall partner, their offspring will inherit genes both for tallness and for preferring tallness. Therefore, the offspring will be genetically predisposed to mate assortatively, without any preference for similarity per se, simply because they will be tall and will prefer tall partners. In this way, assortative mating emerges as a nonadaptive side effect of preferential mating. This genetic-correlation mechanism does not conflict with existing explanations for assortative mating, but it is unique in its simplicity and universality. It suggests that assortative mating is likely to arise naturally in most circumstances, regardless of additional social dynamics or adaptive benefits.

The underlying premise of this genetic-correlation mechanism—that traits and preferences exhibit heritable variation—is well established. Decades of research of identical and nonidentical twins has established that virtually all human traits are heritable (i.e., their variation is due in part to genetic variation; Polderman et al., 2015). Indeed, this observation has been called the *first law of behavioral genetics* (Turkheimer, 2000). The findings of the few behavioral genetic studies of mate preferences are consistent with this observation (Verweij et al., 2012; Zietsch et al., 2015, 2012). Following from this premise, we expect that when individuals mate based on these heritable traits and preferences, genetic correlations form. The notion that preferential mate choice would lead to genetic correlations between preferences and preferred traits is not novel—it is a fundamental concept in the area of mate-preference evolution (Fisher, 1930; Kirkpatrick & Barton, 1997; Lande, 1981). Moreover, twin data has demonstrated these genetic correlations between preferences and preferred traits in humans for height, hair color, intelligence, creativity, an exciting personality, and religiosity (Verweij et al., 2014). The idea that these genetic correlations would then lead to patterns of assortative mating is a key feature in models of speciation (Lande, 1981) and has been sparingly acknowledged in evolutionary psychology (Gangestad, 1989), but has been overlooked in the broader discussion of assortative mating explanations, as evidenced by its absence from recent reviews on the topic (Jiang et al., 2013; Luo, 2017; Versluys et al., 2021).

In this article, we evaluate this genetic-correlation mechanism using an agent-based model in which agents chose reproductive partners based on heritable traits and preferences over 100 generations. We present a model with no selection pressure to investigate the genetic-correlation mechanism in the absence of other dynamics, and we present another model with selection pressure (i.e., on traits through differential reproduction) to assess a more realistic scenario. We present 10 versions of each model to assess how the proposed mechanism was affected by the number of preferences and preferred traits used to evaluate mates (one to ten). At each generation of each model, we display the genetic correlations between traits and corresponding preferences within individuals and the trait correlations between partners (i.e., assortative mating).

## Research Transparency Statement

### General disclosures

**Conflicts of interest:** All authors declare no conflicts of interest. **Funding:** This research received no specific funding. **Artificial intelligence:** No artificial-intelligence-assisted technologies were used in this research or the creation of this article. **Ethics:** This research does not require ethics approval because it does not involve human or other animal participants. **Open Science Framework (OSF):** To ensure long-term preservation, we registered all OSF files at https://doi.org/10.17605/OSF.IO/X7WG3.

### Study disclosures

**Preregistration:** No aspects of the study were preregistered. **Materials:** All study materials are publicly available at https://doi.org/10.17605/OSF.IO/X7WG3. **Data:** All simulated data are publicly available at https://doi.org/10.17605/OSF.IO/X7WG3. **Analysis scripts:** All simulation and analysis scripts are publicly available at https://doi.org/10.17605/OSF.IO/X7WG3. **Computational reproducibility:** The computational reproducibility of the results in the main article (but not the Supplemental Material) has been independently confirmed by the journal’s STAR team.

## Method

We investigated assortative mating using an agent-based model programmed in R. Agent-based modeling is a computational approach that simulates individuals interacting under a specified set of rules (Smaldino, 2020), allowing us in this case to observe how individual mate choices can lead to population-level outcomes over many generations. Agents in the model used ideal preference values to evaluate potential partners’ traits, which were either fitness-neutral (no-selection-pressure model) or were used to implement selection pressure through differential reproduction (selection-pressure model). Each simulation ran for 100 generations, which was long enough to observe correlations between partner traits while minimizing computational load. We ran 10 versions of each model to assess how varying the number of preferences used in mate choice from one to ten affected the strength of correlations between partners’ traits. The following sections describe the agents’ properties and how agents chose partners and reproduced. The full code and simulation results can be found at https://doi.org/10.17605/OSF.IO/X7WG3. This study involved only simulated data and did not include human or animal participants; consequently, no ethical review was required under the policies of The University of Queensland or the relevant Australian bodies.

### Agent properties

An initial population of 300 agents was generated, each representing an individual in the population, with sex randomly determined. The population size of 300 was intended to represent a situation in which agents had many partner options but not infinitely so. Each agent had values for 10 traits and a certain number of preferences (from one to ten) used for mate choice, which was varied across the 10 models.

Traits and preferences were entirely genetically determined with no environmental variance. Each trait and each preference was determined by 40 unique loci; there were no initial associations among traits, among preferences, or between traits and preferences. We used 40 loci to ensure the traits behaved as polygenic quantitative traits, as are virtually all human traits, but without the prohibitive computational demand of larger, more realistic numbers of underlying loci. Each locus had two alleles, which could be one of two variants: 0 or 1. The initial population’s alleles were randomly determined as 0 or 1 with 50% probability. The final trait and preference values were determined by summing all allele values for the contributing loci, meaning that trait and preference values ranged from 0 to 80 with an initial population mean of approximately 40. This toy model provided a simplified representation of the mate-choice mechanism we were investigating, and therefore the genetic and population structure described here was not intended to perfectly match the complex genetic and population structure of humans.

### Determining reproductive partners

#### Determining interest

To determine suitable pairings for reproduction, we first calculated the interest of each agent in all opposite-sex agents. This interest was quantified using the Euclidean distance between each agent’s preference values and the corresponding trait values of potential partners, as detailed in Equation 1. Specifically, Euclidean distance_*i,k*_ is the Euclidean distance between agent *i* and potential partner *k*, preference_*i,j*_ is the preference value of agent *i* for trait *j*, trait_*k,j*_ is the trait value of potential partner *k* for trait *j*, and *n* is the total number of traits. Interest scores between agents who had the same parents (i.e., siblings) were set as null values and were therefore not considered in the processes described hereafter:

(1) $$\begin{array}{c} {\mathrm{Euclidean}\;\mathrm{distance}}_{i,k}=\sqrt{\sum_{j=1}^{n}{\left({\mathrm{preference}}_{i,j}-{\mathrm{trait}}_{k,j}\right)}^{2}} \end{array}$$

Euclidean distance scores were transformed into interest scores by reversing the scale in such a way that high Euclidean distance scores become low-interest scores and low Euclidean distance scores become high-interest scores. This reflects that interest in a potential partner is highest when an agent’s preferences are closest to a potential partner’s traits. This transformation was performed separately for all male agents’ scores and all female agents’ scores using equation (2) below for each agent *i* and potential partner *k*:

(2) $$\begin{array}{c} \begin{array}{l} {\mathrm{interest}}_{i,k}=\min\left(\mathrm{Euclidean}\;\mathrm{distance}\right)\\ +\max\left(\mathrm{Euclidean}\;\mathrm{distance}\right)\;-\;{\mathrm{Euclidean}\;\mathrm{distance}}_{i,k} \end{array} \end{array}$$

Research suggests Euclidean distance is an effective measure for predicting interest in potential partners because it accounts for multiple traits simultaneously, including nonlinear effects (Conroy-Beam & Buss, 2016, 2017). Although metrics like semi-Euclidean distance and pattern matrices perform similarly in predicting long-term relationship outcomes (Driebe et al., 2024), we opted to use Euclidean distance for two reasons. First, Euclidean distance has been specifically demonstrated to effectively predict who pairs with whom, which is most relevant to this model (Conroy-Beam et al., 2022). Second, other measures were incompatible with our model design. Semi-Euclidean distance assumes that higher trait levels are generally considered more desirable, but because our model depicts the evolution of preferences, it does not make sense to assume a universal, preexisting population-level preference for higher trait values. Pattern matrices rely on the relationships among multiple traits and preferences, which would be unsuitable for our one-preference model and might introduce spurious effects across models with differing numbers of preferences used.

#### Forming exclusive pairs

Next, we used the *resource allocation model* to determine which opposite-sex agents would form exclusive pairs on the basis of mutual interest (Conroy-Beam, 2021). This algorithm was developed to emulate the dynamic allocation of limited mating resources to potential partners and has demonstrated high accuracy in identifying real couples. The resource allocation model is an iterative process that we ran for 20 rounds. Each agent had a total resource value of 1, representing the overall time, effort, and other resources they could invest in securing a partner. In the first round, agents distributed their total resources proportionally among all opposite-sex agents on the basis of their initial interest score for each potential partner. These resource allocations were calculated with Equation 3 below for agent *i* and potential partner *k*, where *l* represents each opposite sex potential partner from 1 to *n* (number of opposite-sex agents):

(3) $$\begin{array}{c} {\mathrm{resource}\;\mathrm{allocation}}_{i,k}=\frac{{\mathrm{interest}}_{i,k}}{\sum_{l=1}^{n}{\mathrm{interest}}_{i,l}} \end{array}$$

After all resource allocations were calculated, agents observed the resources allocated to them in return by each potential partner before deciding how to redistribute their resources in the next round. Agents calculated updated interest scores by multiplying their given and received resource allocation for each partner according to Equation 4 below for agent *i* and potential partner *k*:

(4) $$\begin{array}{c} \begin{array}{l} {\mathrm{updated}\;\mathrm{interest}}_{i,k}={\mathrm{resource}\;\mathrm{allocation}}_{i,k}\\ \;\;\times {\mathrm{resource}\;\mathrm{allocation}}_{k,i} \end{array} \end{array}$$

This process drove agents to shift their resources toward potential partners who showed reciprocal investment. The updated interest scores were fed back into Equation 3, beginning an iterative cycle between Equations 3 and 4. This process encourages agents to narrow down their resources from all potential mates to a select few who show mutual interest. After 20 rounds, agents who were both most interested in each other formed exclusive reproductive pairs, whereas those without a reciprocated most-interested partner did not pair.

### Reproduction

#### No-selection-pressure model

All exclusive reproductive pairs within a generation produced the same number of offspring. This number was calculated each generation to maintain a consistent population size and was typically two or three offspring.

#### Selection-pressure model

Each pair was given a fitness score, calculated as the Euclidean distance between each of their traits and the ideal trait value. Based on this score, each pair was assigned a number of offspring sampled from a Poisson distribution with a lambda that was calculated each generation to keep population size consistent (typically 2 or 3). Trait values could theoretically range from 0 to 80, with an initial mean of 40. The ideal trait value was set at 60 to prompt active evolution on traits.

In both models, offspring sex was randomly determined. Alleles for traits and preferences were passed down through independent assortment; at each locus, an offspring randomly inherited one allele from each parent. We implemented a mutation rate of 0.01, giving each allele a 1% chance of toggling between 0 and 1 during inheritance.

## Results

### No-selection-pressure model

**Fig. 1.**
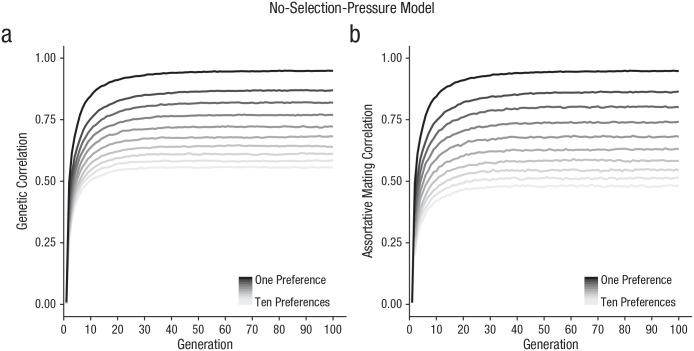
No-selection-pressure model results. In (a), we show genetic correlations between individuals’ traits and corresponding preferences for models varying in how many preferences are used to evaluate mates (ranging from one to ten). In (b), we show partner-trait correlations (i.e., assortative mating) for models varying in how many preferences are used to evaluate mates (ranging from one to ten).

Figure 1a shows the genetic correlations for individuals’ preferences and preferred traits over 100 generations (with preferences ranging from one to ten). Results indicated that genetic correlations formed between preferences and preferred traits through preferential mating, as expected. Figure 1b shows trait correlations between partners over 100 generations for each model, indicating that assortative mating also emerged. The assortative-mating correlations were weaker than the genetic correlations—especially in models with more mate preferences—likely because the constraints involved in multivariate mate choice prevent genetic preferences from being fully realized. The individual genetic correlations and assortative-mating correlations were themselves strongly correlated at each generation (*r* = .989, *p* < .001), consistent with our expectation that assortative mating would be driven by genetic correlations forming between traits and preferences.

### Selection-pressure model

**Fig. 2.**
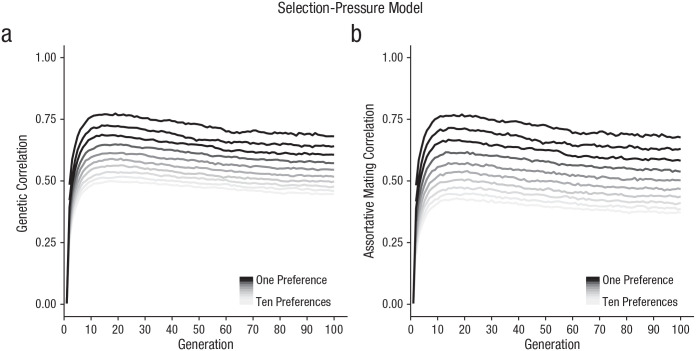
Selection-pressure model results. In (a), we show genetic correlations between individuals’ traits and corresponding preferences for models varying in how many preferences are used to evaluate mates (ranging from one to ten). In (b), we show partner-trait correlations (i.e., assortative mating), for models varying in how many preferences, are used to evaluate mates (ranging from one to ten).

The selection-pressure models demonstrated a pattern of results that was consistent with the models with no selection pressure above, with genetic correlations forming between preferences and preferred traits (Fig. 2a) and assortative mating emerging over generations (Fig. 2b). Again, the assortative mating correlations tended to be weaker than the genetic correlations. The two correlation measures were themselves strongly correlated at each generation (*r* = .975, *p* < .001), consistent with our expectation that assortative mating would be driven by genetic correlations forming between traits and preferences.

### Nonheritable-preferences model

To ensure that heritable variation in preferences and preferred traits was the driving force of assortative mating in our models, we also ran the no-selection-pressure model with zero heritability for preferences (i.e., preference values were sampled at random from a distribution matching the no-selection-pressure model). Under this condition, we observed no correlations between individuals’ preferences and preferred traits, and no assortative mating emerged (further details are available in the Supplemental Material available online). There was still a strong association between the genetic correlations and assortative mating correlations (*r* = .816, *p* < .001), indicating that even chance fluctuations in correlations between individual preferences and preferred traits influenced assortative mating, consistent with the genetic-correlation mechanism.

## Discussion

Assortative mating is a ubiquitous phenomenon observed in both human and animal mating (Horwitz et al., 2023; Jiang et al., 2013), for which numerous explanations have been proposed (Versluys et al., 2021). Here, we presented an overlooked genetic mechanism: Assortative mating is a natural consequence of heritable variation in preferences and preferred traits. When individuals mate according to heritable preferences and preferred traits, genetic correlations form between the two (Fisher, 1930; Kirkpatrick & Barton, 1997; Lande, 1981; Verweij et al., 2014). It follows that individuals would prefer and choose partners whose traits are similar to their own, because preferring a trait is associated with having that same trait (Gangestad, 1989; Lande, 1981). In this way, assortative mating is expected to emerge as a nonadaptive side effect of preferential mating.

Our agent-based model demonstrated that even with up to 10 preferences used in mate choice, substantial genetic correlations formed between traits and corresponding preferences, resulting in assortative mating. Heritable variation in preferences and traits alone was sufficient to drive this pattern, in the absence of more complex dynamics often emphasized in this field. In the models with selection pressure on the traits, all correlations appeared less stable and were slightly lower than in the models with no selection pressure, likely because selection pressure reduces variance in traits. We do not believe that selection pressure introduced new dynamics affecting the evolution of assortative mating beyond this variance-reducing effect.

### Limitations

Although our model successfully demonstrated the genetic-correlation mechanism, it is a limited representation of reality. The model represents a simplified, toy model of genetics that is not intended to fully capture the complex nature of real genetic architecture. Additionally, the generalizability of our findings is limited by our model’s focus on human mating patterns, including mutual mate choice, monogamy, and the specific partner-selection algorithms we employed (Conroy-Beam, 2021; Conroy-Beam & Buss, 2017). Nevertheless, these factors are unlikely to alter the conclusion that assortative mating naturally arises from heritable variation in traits and preferences. For instance, we also ran a model of a lekking mating system, in which only females exhibit mate choice and males mate with any female that chooses them (see the Supplemental Material); we found that assortative mating emerged with a similar pattern of results.

Traits for which entirely different genetic factors influence trait variation in males and females would not be subject to the genetic-correlation mechanism, as male trait alleles would correlate with female preference alleles and vice versa, preventing the manifestation of a correlation between trait and preference alleles within an individual. However, in reality, largely the same genetic factors influence traits in both sexes for humans, with cross-sex genetic correlations almost always positive and usually close to 1 (Bernabeu et al., 2021; Vink et al., 2012).

We recognize that the genetic-correlation mechanism may not be the sole or primary explanation for assortative mating on any given trait. For instance, the mechanism does not account for assortative mating based on nongenetic factors, such as age. Furthermore, the substantial variation in assortative-mating correlations across different traits suggests the involvement of additional factors beyond the genetic-correlation mechanism. If the genetic correlations between traits and preferences we outlined here were the only cause of assortative mating, we would expect a strong association between the heritability of traits and preferences and the extent of assortative mating. Yet this was not necessarily observed; many traits with lower heritability, such as social attitudes and values, show stronger partner correlations than highly heritable traits like height (Horwitz et al., 2023; Luo, 2017).

Our model does not compare the strength of the genetic-correlation mechanism to other proposed explanations for assortative mating, and its coarse-grained nature does not allow us to quantify how much variance in assortative mating the mechanism might explain. Nevertheless, we offer a brief comparison of values here. First, averaged over generations 50 to 100, our simulations produced genetic-correlation estimates between traits and preferences ranging from 0.46 to 0.95, compared with empirical estimates of 0.27 to 1.00 (Verweij et al., 2014). Our simulations also yielded assortative-mating correlations from 0.38 to 0.95, compared with empirical ranges of around 0.7 to 0.9 for age, 0.4 to 0.6 for education, 0.2 for height, and 0.1 for personality (Horwitz et al., 2023; Luo, 2017). Because our models assume that traits are entirely genetically determined with no measurement or perception error, the resulting correlations are undoubtedly inflated compared with reality. Previous models focused on mating-market dynamics have reported assortative mating correlations of 0.44 to 1.00 (Xie et al., 2015) and 0.55 (Kalick & Hamilton, 1986). The breadth of these estimates highlights the limitations of coarse-grained models in evaluating the true effect size of such mechanisms. More fine-grained models, informed by empirical data, would be necessary to explore the relative contributions of various mechanisms.

It is also unclear how robust these genetic correlations would be in reality. There is debate around whether such genetic correlations are likely to be overwhelmed by direct costs or benefits to mate preferences (Fry, 2022; Servedio, 2024). Additionally, genetic correlations that form through linkage disequilibrium, like the ones in our models, rather than through pleiotropy, in which two traits are contributed by the same alleles, are tenuous. Such genetic correlations could deteriorate within just a few generations when the relevant force is removed—for example, under random mating (Hosken & Wilson, 2019; Roff, 1997).

### Conclusion

The genetic-correlation mechanism presented here is unique in explaining why assortative mating is so widely observed. We have demonstrated that even in the absence of adaptiveness or complex social dynamics, assortative mating is likely to arise naturally when preferences and preferred traits are heritable, which is true for virtually every quantitative trait. We have presented a limited model here, so we cannot speak to the resilience of such genetic correlations in the context of direct costs on mate choice, nor the absolute strength of assortative mating that can be explained by this mechanism compared with other mechanisms. Existing explanations are likely to apply to certain traits under certain conditions, but the genetic-correlation mechanism offers a powerful explanation for the ubiquity of assortative mating across traits, contexts, and species.

## Supplemental Material

sj-docx-1-pss-10.1177_09567976251365900 – Supplemental material for Assortative Mating Is a Natural Consequence of Heritable Variation in Preferences and Preferred Traits Supplemental material, sj-docx-1-pss-10.1177_09567976251365900 for Assortative Mating Is a Natural Consequence of Heritable Variation in Preferences and Preferred Traits by Kaitlyn T. Harper and Brendan P. Zietsch in Psychological Science

## Transparency

*Action Editor:* Julia Stern

*Editor:* Simine Vazire

*Author Contributions*

**Kaitlyn T. Harper:** Conceptualization; Formal analysis; Investigation; Methodology; Software; Visualization; Writing – original draft; Writing – review & editing.

**Brendan P. Zietsch:** Conceptualization; Investigation; Methodology; Supervision; Writing – review & editing.

**Artificial Intelligence:** No artificial-intelligence-assisted technologies were used in this research or the creation of this article.

**Ethics:** This research does not require ethics approval because it does not involve human or other animal participants.

**Open Practices:** Preregistration: No aspects of the study were preregistered. Materials: All study materials are publicly available at https://doi.org/10.17605/OSF.IO/X7WG3. Data: All simulated data are publicly available at https://doi.org/10.17605/OSF.IO/X7WG3. Analysis scripts: All simulation and analysis scripts are publicly available at https://doi.org/10.17605/OSF.IO/X7WG3. Computational reproducibility: The computational reproducibility of the results in the main article (but not the Supplemental Material) has been independently confirmed by the journal’s STAR team. To ensure long-term preservation, we registered all Open Science Framework files at https://doi.org/10.17605/OSF.IO/X7WG3.
