# Supplementary material for: Assortative Mating Is a Natural Consequence of Heritable Variation in Preferences and Preferred Traits
Source: Psychol Sci. 2025 Sep 4;36(10):771–9. doi: 10.1177/09567976251365900 (PMC13428944; doi:10.1177/09567976251365900)
Supplement: Supplementary file 1 [file sj-docx-1-pss-10.1177_09567976251365900.docx]

**SUPPLEMENTARY MATERIAL**

**Assortative Mating is a Natural Consequence of Heritable Variation in Preferences and Preferred Traits**

# Additional model with zero preference heritability

We developed this additional model, where agents’ preferences were not heritable, to verify that no other dynamics were influencing assortative mating in our main model. Specifically, if assortative mating persisted in this additional model when the proposed mechanism—heritable traits and preferences—was disrupted, it would indicate that another factor, beyond our mechanism, was responsible for assortative mating in our main model.

**Model details and results**

In this additional model, agents' preferences were not genetically determined, but were randomly sampled from a distribution that matched the preference distribution in the main model. We did not include selection pressure in this model. All other processes related to mate choice and reproduction remained as described in the main manuscript. Our method of calculating partner trait correlations (i.e. assortative mating) was unchanged, while individual correlations were analysed using agents' trait scores (genetically determined) and their corresponding, randomly determined preference scores.

Figure S1 shows the correlations for individuals’ preferences and preferred traits over 100 generations for each model (with one to ten preferences used in mate choice). Results indicated that no correlations formed between individuals’ preferences and preferred traits. Figure S2 shows trait correlations between partners over 100 generations for each model, indicating that assortative mating did not emerge. There was still a strong association between the individual correlations and partner trait correlations at each generation, *r* = .816, *p* < .001, indicating that even chance fluctuations in correlations between individual preferences and preferred traits influenced assortative mating, consistent with our proposed mechanism. These results confirm that assortative mating in our main model is driven by heritable variation in preferences and preferred traits.

**Figure S1.** Zero heritability model correlations between individuals’ traits and corresponding preferences for models varying in how many preferences are used to evaluate mates (one through to ten).

**Figure S2.** Zero heritability model partner trait correlations (i.e. assortative mating) for models varying in how many preferences are used to evaluate mates (one through to ten).

*Note.* In both figures, the y-axis has been centred around zero to ensure negative values are visible, but the overall y-axis range and scaling remain consistent with graphs in the main manuscript, with a total range of 1.0.

# Additional model with lekking mating structure

We developed this additional model, with a lekking mating structure, to assess whether the genetic correlations mechanism persists in a different, common mating system. If so, this would suggest that this explanation applied broadly across species.

**Model details and results**

Rather than forming exclusive pairs using the Resource Allocation Model, we used a female choice lekking structure. We included selection pressure in the model because it had minimal influence and enhanced realism. All other processes in the model remained as described in the main manuscript, but we replace the “forming exclusive pairs” section with the following: To determine which agents would form reproductive pairs, each female agent simply chose the male agent who she gave the highest interest score. If multiple males tied for her maximum interest, she picked one at random. This approach allowed every female to reproduce once with her chosen partner, while males could mate with multiple females if selected more than once.

Figure S3 shows the correlations for individuals’ preferences and preferred traits over 100 generations for each model (with one to ten preferences used in mate choice). Results indicated that genetic correlations formed between preferences and preferred traits through preferential mating, as expected. Figure S4 shows trait correlations between partners over 100 generations for each model, indicating that assortative mating also emerged. The individual genetic correlations and assortative mating correlations were themselves strongly correlated at each generation (*r* = .978, *p* < .001), consistent with our expectation that assortative mating would be driven by genetic correlations forming between traits and preferences. These results confirm that assortative mating can be driven by heritable variation in preferences and preferred traits, even in lekking mating systems.

**Figure S3.** Lekking model correlations between individuals’ traits and corresponding preferences for models varying in how many preferences are used to evaluate mates (one through to ten).

**Figure S4.** Lekking model partner trait correlations (i.e. assortative mating) for models varying in how many preferences are used to evaluate mates (one through to ten).
